# Circulating microRNAs as predictive biomarkers of coronary artery diseases in type 2 diabetes patients

**DOI:** 10.1002/jcla.24380

**Published:** 2022-03-29

**Authors:** Golnoosh Mahjoob, Yasin Ahmadi, Huda Fatima rajani, Nafiseh khanbabaei, Sakhavat Abolhasani

**Affiliations:** ^1^ Department of Clinical Biochemistry Sarab Faculty of Medical Sciences. Sarab Iran; ^2^ Department of Clinical Biochemistry Tarbiat Modares University Tehran Iran; ^3^ 449486 Department of Medical Laboratory Sciences College of Science Komar University of Science and Technology Sulaimani Iraq; ^4^ Department of medical biotechnology School of advanced sciences in medicine Tehran University of medical sciences Tehran Iran

**Keywords:** biomarker, coronary artery diseases (CAD), diabetes mellitus type 2 (T2DM), microRNAs

## Abstract

**Background:**

Type 2 diabetes mellitus (T2DM) is an increasing metabolic disorder mostly resulting from unhealthy lifestyles. T2DM patients are prone to develop heart conditions such as coronary artery disease (CAD) which is a major cause of death in the world. Most clinical symptoms emerge at the advanced stages of CAD; therefore, establishing new biomarkers detectable in the early stages of the disease is crucial to enhance the efficiency of treatment. Recently, a significant body of evidence has shown alteration in miRNA levels associate with dysregulated gene expression occurring in T2DM and CAD, highlighting significance of circulating miRNAs in early detection of CAD arising from T2DM. Therefore, it seems crucial to establish a link between the miRNAs prognosing value and development of CAD in T2DM.

**Aim:**

This study provides an overview on the alterations of the circulatory miRNAs in T2DM and various CADs and consider the potentials of miRNAs as biomarkers prognosing CADs in T2DM patients.

**Materials and Methods:**

Literature search was conducted for miRNAs involved in development of T2DM and CAD using the following key words: “miRNAs”, “Biomarker”, “Diabetes Mellitus Type 2 (T2DM)”, “coronary artery diseases (CAD)”. Articles written in the English language.

**Result:**

There has been shown a rise in miR‐375, miR‐9, miR‐30a‐5p, miR‐150, miR‐9, miR‐29a, miR‐30d, miR‐34a, miR‐124a, miR‐146a, miR‐27a, and miR‐320a in T2DM; whereas, miR‐126, miR‐21, miR‐103, miR‐28‐3p, miR‐15a, miR‐145, miR‐375, miR‐223 have been shown to decrease. In addition to T2DM, some miRNAs such as mirR‐1, miR‐122, miR‐132, and miR‐133 play a part in development of subclinical aortic atherosclerosis associated with metabolic syndrome. Some miRNAs increase in both T2DM and CAD such as miR‐1, miR‐132, miR‐133, and miR‐373‐3‐p. More interestingly, some of these miRNAs such as miR‐92a elevate years before emerging CAD in T2DM.

**Conclusion:**

dysregulation of miRNAs plays outstanding roles in development of T2DM and CAD. Also, elevation of some miRNAs such as miR‐92a in T2DM patients can efficiently prognose development of CAD in these patients, so these miRNAs can be used as biomarkers in this regard.

## Graphical abstract

There is a considerable number of miRNAs in T2DM leading to CAD and its complications. Furthermore, miRNAs represent potential novel therapeutic targets for several cardiovascular disorders. This study provided an overview of the alterations in circulatory miRNAs in T2DM and various CADs.

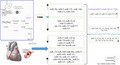

## 1 INTRODUCTION

About half a billion people across the globe are affected by diabetes. This disease predisposes a significant number of people to life‐threatening complications such as heart conditions and other diabetes‐related morbidities.^1^

Sedentary lifestyles, obesity, aging, and urbanization are the contributing factors for type 2 diabetes mellitus (T2DM). Additionally, hereditary backgrounds are involved in the etiology of the disease.^2^ T2DM is characterized by chronic hyperglycemia and faulty metabolism of carbohydrates, lipids, and proteins, resulting from insulin resistance and insufficient insulin secretion.^3^ Regarding dysregulated metabolism of lipids, patients with T2DM are highly susceptible to cardiovascular diseases, particularly coronary syndrome.^4^

Acute coronary syndrome (ACS) is an expression to describe three types of coronary artery disease due to the dissociation of plaques from the lumen of the coronary artery. The classification is based on the location of the blockage, duration of blockage, and severity of damage. These life‐threatening conditions require urgent medical intervention. The three types are unstable angina, non‐ST segment elevation myocardial infarction (NSTEMI), and ST‐segment elevation myocardial infarction (STEMI). Unstable Angina is the acute form of coronary syndrome and can simply turn into myocardial infarction. As a result, it is treated as a medical emergency. Non‐ST segment elevation myocardial infarction (NSTEMI) may not cause changes on an electrocardiogram (ECG). In addition, the blockage may be partial or temporary, and so the extent of the damage is relatively small. However, ST‐segment elevation myocardial infarction (STEMI) arises from the prolonged blocked blood supply.^5^

Establishing simple and valid clinical biomarkers is crucial in detecting and treatment of different coronary artery diseases (CAD) and T2DM. microRNAs (miRNAs) can be used as biomarkers which are highly stable in circulation and specific to disease and organ; thus, they have potential to be applied as reliable diagnostic biomarkers.^6^

miRNAs are stable single‐stranded RNAs modulating various biological processes by regulating gene expression via binding to the 3’‐ untranslated regions of target mRNAs at the post‐transcriptional level.^7^ They are primarily located within the introns of the host genes and are transcribed by RNA polymerase II.^8^, ^9^

The human genome encodes roughly 1000 miRNAs, more than 100 of them have been identified in the serum of healthy subjects. Unlike intracellular mRNAs, circulating miRNAs are significantly resistant against degradation by RNases. These miRNAs may also be produced by blood cells or tissues like heart, lung, liver, and kidney.^6^ They are mostly preserved in macrovesicles such as exosomes, microparticles, and apoptotic bodies, possibly protecting them against degradation by RNases. Their stability and source specificity make miRNAs as reliable candidates in detecting diseases like CAD and T2DM.^6^, ^10^

## 2 ROLES OF miRNAs IN T2DM DEVELOPMENT

Dysregulation of miRNAs has been demonstrated widely in T2DM (see Table 1). There is a rise in, miR‐9, miR‐30a‐5p, miR‐150, miR‐9, miR‐29a, miR‐30d, miR‐34a, miR‐124a, miR‐146a, miR‐27a, and miR‐320a in T2DM. On the contrary, other miRNAs such as miR‐126, miR‐21, miR‐103, miR‐28‐3p, miR‐15a, miR‐145, miR‐375, and miR‐223 were shown to be decreased.^11^, ^12^

**TABLE 1:** Changes in the levels of miRNAs in T2DM

Upregulation	Downregulation
miR−9,^17^ miR−30a−5p,^19^ miR−150,^20^ miR−29a,^21^ miR−30d,^14^ miR−34a,^22^ miR−124a,^23^ miR−146a,^15^ miR−27a,^23^ miR−320a^24^	miR−126,^24^ miR−21,^25^ miR−103,^26^ miR−28‐3p z24), miR−15a,^27^ miR−145,^28^ miR−375,^29^ miR−223^30^

Kong et al. studied the expression pattern of several miRNAs associated with the pathogenesis of T2DM and compared the results among the recently diagnosed cases of T2DM, pre‐T2DM subjects, and T2DM‐susceptible subjects with normal glucose tolerance.^13^ They showed the levels of miRNAs were higher in T2DM patients compared to T2DM‐susceptible subjects. Additionally, the expressions of some miRNAs were significantly lower in the pre‐T2DM individuals in comparison with T2DM patients. This study also demonstrated the role of miR‐29a, miR‐9, miR‐30d, miR‐124a, miR‐146a, miR‐34a, and miR‐375 in fine‐tuning insulin secretion and pathogenesis of T2DM, while their expression levels remained steady in the pre‐T2DM stage, refusing their potential relevance as disease‐specific markers.^14^

The role of miR‐146a in the inflammation and insulin resistance was shown to be more significant in patients with T2DM than in those with normal glucose tolerance (NGT). There was a significant lower expression level of miR‐146a in T2DM subjects.^15^

Further association between miRNAs and metabolic syndrome was demonstrated; mirRNA‐150, miR‐192, miR‐27a, miR‐320a, and miR‐375 were upregulated in T2DM, highlighting their part in the regulation of hyperglycemia. This finding made great strides in introducing the clinical application of these miRNAs in the risk assessment of T2DM and metabolic syndrome.^16^

Zhang et al. assessed the miRNA in individuals with normal blood glucose, individuals susceptible to developing T2DM, and T2DM patients; they showed only miR‐15a, miR‐223, and miR‐126 were detectable in T2DM patients. A significant finding was that the plasma miR‐126 levels were similar between pre‐T2DM and T2DM groups, while, it was substantially lower in the NGT group. Furthermore, the serum levels of miR‐126 were shown to be associated with levels of fasting glucose in the serum. This study suggested that the plasma miR‐126 could be regarded as a biomarker for the non‐invasive prediction and diagnosis of T2DM.^17^

Stepien et al. analyzed the angiogenic capacity of ectosome‐derived miRNAs among T2DM patients. They reported that miR‐221‐3p, miR‐20a‐3p, let‐7i‐5p miR‐26a‐5p, miR‐26b‐5p, miR‐30b‐5p miR‐29a‐5p, miR‐374a‐5p, miR‐30c‐5p, and miR‐199a‐3p were significantly elevated in ectosomes obtained from T2DM patients [57]. Also, they showed that the expression levels of miR‐193b‐3p and miR‐95‐3p in the ectosomes‐enriched plasma were notably higher in T2DM, while the expression of miR‐409‐3p was lower in T2DM. These findings may highlight the role of miRNAs in dysregulated angiogenesis and development of vascular complications in patients with T2DM.^18^ Table 1 shows the status of different miRNAs in T2DM.

## 3 ROLES OF miRNA IN CAD

Micro‐RNAs play a critical role in cardiac development and pathological processes such as AMI (including NSTEMI and STEMI) and other cardiovascular diseases such as arrhythmias, hypertrophy, heart failure, and atherosclerosis.^31^ More than 200 miRNAs have been identified in the heart tissues. miRNAs such as miR‐1, let‐7, miR‐133, miR‐126‐3p, miR‐30c, and miR‐26a were found to be prevalent in the cardiac muscles and miR‐145, let‐7, miR‐125b, miR‐125a, miR‐23, and miR‐143 in the arterial smooth muscles.^32^, ^33^ Additionally, miR‐122 and miR‐1 have been introduced as myocardium‐specific markers.^34^

Alterations in some miRNAs can be used as diagnostic biomarkers for coronary artery diseases, such as the downregulation of miR‐378, 196‐5p, and 3163‐3p, which can assist in distinguishing CAD patients from normal subjects.^35^, ^36^ While upregulation of miR‐223 shows the occurrence of CAD.^37^

There has been shown a reduction in the miR‐15 family in MI which can be used in diagnosis of T2DM patients and pre‐diabetic ones.^38^ In addition, several miRNAs in patients with ST‐segment elevation MI, miR‐142‐3p and miR‐17‐5p could be considered as potential diagnostic markers.^39^

The prognostic value of miR‐1254 was shown. This micro‐RNA correlates with the left ventricular remodeling and systolic function.^39^

Also, circulating miR‐22‐5p and miR‐150‐3p were overexpressed in the early stage of acute MI. On the other hand, miR‐132‐5p was reduced in patients with acute MI.^40^, ^41^

Moreover, miR‐499 has previously been used in the diagnosis of AMI and NSTEMI whereas miR‐423 was used in the diagnosis of AMI and STEMI.^42^ Early detection of AMI has been facilitated via measuring miR‐208 only one hour after infarction. However, its levels fell after three hours. In such a case, assessment of other markers including miR‐499 and miR‐133 is crucial.^43^

The analysis of patients with both stable CAD and acute coronary syndrome showed upregulation of miR‐92a‐3p and miR‐206^44^, ^45^; while reduced levels were observed in miR‐939 and miR‐181‐a‐3p and 181‐a‐5p..^13^, ^46^

## 4 miRNAs RELEASED BY VASCULAR AND BLOOD CELLS CAN BE USED AS BIOMARKERS CAD DEVELOPMENT

CAD stems from atherosclerosis, an inflammatory disease of the arteries characterized by the formation of atherosclerotic plaques. These plaques cause the narrowing of the lumen of the artery, which can lead to thrombus formation and eventually complete obstruction of the lumen. All the cellular components involved in atherosclerosis are susceptible to release miRNAs into the bloodstream. On the other hand, some specific miRNAs may be taken up by the cells involved in atherosclerosis. In both conditions, miRNAs can be considered as diagnostic biomarkers of atherosclerosis.^47^ Various miRNA including miR‐181d, miR‐140‐3p, miR‐182, miR‐145, miR‐19a, miR‐584, miR‐155, miR‐222, miR‐29a, miR‐378, miR‐342, miR‐150, and miR‐30e‐5p in whole blood,^48^ and miR‐126, miR‐92a, miR‐145, miR‐17, and miR‐155 in in EDTA (ethylenediaminetetraacetic acid) plasma and serum have been shown as the biomarkers of CAD.^47^, ^48^ The prognostic and diagnostic value of miRNAs is shown in Table 2.

**TABLE 2:** Roles of CAD‐related miRNAs in diagnosis, severity, and prognosis of heart diseases

Disease	miRNA alterations		Diagnosis		Ref.
	Increase	Decrease	increase	decrease	
Unstable angina	miR−135 and miR147	miR−135, 147	miR−122‐5p, 223‐3p, 941	miR−378, 196‐5p, 3163‐3p	49, 50, 51, 52
Acute MI	miR−1, 122, 126‐3p, 30c, 26a, miR−22‐5p and miR−150‐3p	miR−15 family, 195, miR−590‐3p, miR−199a−3p, miR−132‐5p	miR−223	miR−378, 196‐5p,3163‐3p	17, 18, 53, 54
NSTEMI	miR−221‐3p, 483‐5p		miR−223	miR−378, 196‐5p,3163‐3p	53, 55
STEMI	miR−221‐3p, 374b−5p		miR−142‐3p, miR−17‐5p, 499‐a−5p,223	miR−378,196‐5p,3163‐3p	35, 56, 57
HF	miR−499, 423‐5p, 21, 132, 122, 210, 499, 622	miR−150‐5p	miR−223	miR−378,196‐5p,3163‐3p	58, 59, 60
Arrhythmia	miR−1443p,145‐5p, 185‐5p, 494	miR−23a and miR−26a	miR−223	miR−378,196‐5p,3163‐3p	58, 60, 61
Atherosclerosis	miR−133, 208a, 146b,34a, 21, 30a, 106b, 133a, 208a, 155, 223	miR−31, 720, 126‐5p, 17–92	miR−223	miR−378,196‐5p,3163‐3p	62, 63, 64

Abbreviations: HF, Heart Failure; MI, Myocardial Infarction; NSTEMI, non‐ST‐segment elevation myocardial infarction; STEMI, ST‐elevated myocardial infarction.

Fichtlscherer et al. measured 8 miRNAs in patients with CAD; the endothelial enriched miRNAs including miR‐126, miR‐17, and miR‐92a, the smooth‐muscle cell‐enriched miR‐145, and the inflammatory cell‐enriched miR‐155 were significantly reduced, whereas the cardiomyocyte enriched miRNAs miR‐133 and miR‐208a were elevated.^65^ Also, elevations in miR‐146, miR‐19, miR‐155, miR‐21, as well as miR‐223 were shown in patients with ACS compared to patients with CAD.^66^ Afterward, Li et al. identified miR‐130a, miR‐21, miR‐27b, and miR‐210 as potential biomarkers for peripheral artery disease. The promising finding of this study shows a lack of significant overlap between the elevated miRNAs in coronary and peripheral atherosclerosis. Accordingly, this finding may indicate the tissue‐specificity of these miRNAs.^67^

Taurino et al. assessed the potential of circulating miRNAs in whole blood as biomarkers for CAD; they showed an elevation in the levels of miR‐140‐3p and miR‐182 in CAD patients.^48^ Also, lower levels of miR‐19a, miR‐181d, miR‐222, miR‐342, miR‐484, miR‐155, miR‐145, miR‐29a, miR‐378, miR‐150, and miR‐30e‐5p were shown in CAD patients.^61^, ^65^

Peripheral blood mononuclear cells have been shown to release miRNA biomarkers specific to acute coronary syndromes and unstable angina. The miRNA signature was studied by Hoekstra et al. in PBMCs of patients with CAD; they revealed that miR‐135 and miR147 were downregulated in patients with stable and unstable angina pectoris (AP). Also, they showed that three miRNAs (miR‐134, miR‐370, and miR‐198) were remarkably upregulated in the PMNCs of patients with unstable AP compared with stable AP, suggesting that the expression of these miRNAs in PMNCs could be regarded as a risk factor of developing acute coronary syndromes.^68^ Furthermore, Takahashi et al. showed the elevated levels of the miRNAs associated with inflammation (miR‐146a and miR‐146b) in PBMCs of stable patients with CAD. After 12 months, 19 percent of the patients experienced a cardiac event; thereby, miR146a level turned out to be a useful independent prognostic marker of these events. Furthermore, in circulating endothelial progenitor cells, miR‐221 and miR‐222 were found to be elevated in patients with CAD compared to those with no indication of CAD.^69^

## 5 microRNA IN T2DM PREDICTING CAD

Diabetic heart disease (DHD) is defined as the heart disease in diabetic individuals including CAD, heart failure, and/or cardiomyopathy.^70^ It mostly arises from obesity, physical inactivity, advanced age, and metabolic syndrome.^71^, ^72^, ^73^, ^74^, ^75^ In a study performed by Ramzan et al., miR‐15a‐5p, miR‐17‐5p, miR‐370‐3p, and miR‐375 significantly predicted metabolic syndrome. Analysis of predictive miRNAs showed miR‐15a‐5p and miR‐17‐5p. were involved in the regulation of metabolic pathways, including insulin, *wnt*, fatty acid metabolism, and *AMPK*.^76^

A study of the expression of miR‐126 and miR‐26a showed significant differences between patients with and without T2DM. Both miRNAs were downregulated in T2DM patients. Interestingly, patients with less miR‐26a and miR‐126 were more prone to develop subsequent CAD. Also, miR‐24 was shown to reduce in T2DM‐CAD patients, while the mRNA of YKL‐40, an inflammatory mediator involved in endothelial dysfunction, was elevated in both T2DM‐CAD and CAD patients.^77^ Furthermore, it was reported that the reduced levels of miR‐145, miR‐9, miR‐15a, miR‐103, miR‐28‐3p, miR‐29a, miR‐223, miR‐126, and miR‐375 are reliable predictors of CAD patients in type 2 diabetes. miR‐24 and its target chitinase 3‐like 1 (Chi3l1/YKL40) were also reduced in type 2 diabetes patients with CAD.^77^, ^78^ Additionally, hyperglycemic‐induced alterations in miRNA in patients with diabetic heart issues are likely to be irreversible even after glycemic control.^79^

The levels of miR‐92a in diabetes patients were shown to precede the emerging of acute coronary at least 2 years before. This study showed that these T2DM patients had a significant increase in the levels of miR‐92a compared to coronary heart disease. Also, this study indicated that miR‐92a is associated with the coincidence of diabetes and heart disease, as well as high blood pressure and HbA1c.^80^ It is also implicated that miR‐133a and miR‐373 mediate signaling of myocytes enhancer factor2C (MEF2C) in diabetic cardiomyopathy, which is an essential transcription factor underlying myocardial hypertrophy and cardiac fibrosis.^81^, ^82^

Table 3 shows miRNAs involved in metabolic syndrome with similar roles in T2DM and CAD.

**TABLE 3:** The role of miRNAs in metabolic syndrome in T2DM and CAD

miRNAs as T2DM biomarkers	Glucose metabolism	Inflammation	Platelet reactivity	Endothelial dysfunction	Overlapped change in miRNAs in both CAD and T2DM
Upregulation	miR−124a,^23^ 150,^83^ 27a,^23^ 30a−5p,^19^ 30d,^21^ 320a,^16^ 34a,^16^ 146a^84^	miR−146a^85^			miR−1,^86^, ^87^, ^88^ miR−132, miR−133,^86^ miR−373–3‐p^89^
Downregulation	miR−375,^90^ 9,^91^ 29a,^92^ 103,^26^ 21,^93^ 28‐3p,^24^ 223,^30^ 145,^28^ 15a,^27^ 126^24^	miR−103,^26^ 21‐5p,^58^ 126‐3p^94^	miR−223,^95^ 140,^63^ 26b, 126^95^	miR−21‐5p,^58^ 126‐3p, 26a,^96^ 24^61^	

## 6 CLINICAL PERSPECTIVE AND CONCLUSION

Since their identification, cardiovascular miRNAs have always been regarded as key players in regulating cardiac gene expression under normal and pathological conditions.^97^ Since T2DM patients are highly susceptible to develop CAD, so establishing reliable biomarkers to prognosis CAD in these patients can play a significant part in mitigation T2DM burden.

Dysregulation of miRNAs occurs in T2DM and CAD and underlying pathological events in both situations (see Tables 1,2, and 3). A pile of studies has shown that miR‐1, miR‐132, miR‐133, and miR‐373–3‐p increase in both T2DM and CAD (see Table 3). Some of these miRNAs emerge long before cardiac events. For instance, the levels of miR‐92a in acute coronary patients were shown to be preceded by diabetes for at least two years.^80^ Accordingly, these miRNAs may assist in following up T2DM patients who are prone to develop CAD.

Regarding the Role of miRNAs in atherosclerosis which is the primary cause of CAD, it is reported that microRNAs participate either beneficially or harmfully in almost all molecular pathways of atherosclerosis and arterial remodeling, including endothelial dysfunction, monocyte activation, arterial wall invasion, platelet, and vascular smooth muscle cell activation.^98^ Atherosclerosis has been regarded as a disease of chronic inflammation.^99^, ^100^ Therefore, miRNA may play their part in heart‐related diseases through induction of inflammation. miR‐92a has been shown to contribute to cardiovascular disease development in diabetes mellitus through NF‐κB and downstream inflammatory pathways.^101^ Also, miR‐132 was shown to inhibit the expression of SIRT1 and induce the pro‐inflammatory processes of vascular endothelial inflammation through blocking the SREBP‐1c metabolic pathway.^102^ mir‐1, miR‐122, miR‐132, and miR‐133 are related to subclinical aortic atherosclerosis associated with metabolic syndrome.^103^ Therefore, these miRNAs may strongly prognose the development of CAD in T2DM patients.

Along with miRNAs, the inflammatory biomarkers can be used as predictors of severity and prognosis in CAD in T2DM patients to stratify the risk of these patients, to take the best therapeutic approach, and predict the results after interventions.^99^

Some limitations should be regarded in future studies. Most studies have evaluated miRNAs in populations of fewer than 100 subjects, so bigger sample sizes seem to be crucial in future studies. Also, the value of the miRNAs in the prognosis of the diseases, such as the risk of MI, should be assessed. Furthermore, it would clarify whether miRNAs levels are practical tools to evaluate the response to therapy,.^104^ Also, low levels of total RNA in plasma or serum, which make the miRNA amplification often necessary to measure circulating miRNAs. U6 RNA or other miRNAs have been used as internal controls. While these miRNAs may be steady in several cases, in other pathological conditions they may fluctuate; therefore, they are not reliable as an internal control.^10^

## CONFLICT OF INTEREST

The authors declare no conflict of interest.

## AUTHOR CONTRIBUTIONS

Conception and design of the research and obtaining financing: Abolhasani S; supervising the team: Golnoosh Mahjoob; preparing and writing of the manuscript: Yassin Ahmadi; critical revision: Huda Fatima Rajani; participated in preparation of the manuscript: Khanbabaei N.

## Data Availability

The datasets collected and analyzed during the current study are available from the corresponding author on reasonable request. Furthermore, the name of repositories and reference numbers can be found in online repositories.

## References

[1] Arora A , Behl T , Sehgal A , et al. Unravelling the involvement of gut microbiota in type 2 diabetes mellitus. Life Sci. 2021;273:119311. 33662428 10.1016/j.lfs.2021.119311

[2] Federation ID . IDF Diabetes Atlas. 7th edn., 2015.

[3] Wang S , Yong H , He XD . Multi‐omics: opportunities for research on mechanism of type 2 diabetes mellitus. World J Diabetes. 2021;12(7):1070. 34326955 10.4239/wjd.v12.i7.1070 PMC8311486

[4] Martín‐Timón I , Sevillano‐Collantes C , Segura‐Galindo A , Del Cañizo‐Gómez FJ . Type 2 diabetes and cardiovascular disease: have all risk factors the same strength? World J Diabetes. 2014;5(4):444‐470. 25126392 10.4239/wjd.v5.i4.444 PMC4127581

[5] Simons M , Alpert J , Cannon C . Acute coronary syndrome: terminology and classification. UpToDate Com. 2020.

[6] Chen X , Ba Y , Ma L , et al. Characterization of microRNAs in serum: a novel class of biomarkers for diagnosis of cancer and other diseases. Cell Res. 2008;18(10):997‐1006. 18766170 10.1038/cr.2008.282

[7] Lewis BP , Shih IH , Jones‐Rhoades MW , Bartel DP , Burge CB . Prediction of mammalian microRNA targets. Cell. 2003;115(7):787‐798. 14697198 10.1016/s0092-8674(03)01018-3

[8] Bagga S , Bracht J , Hunter S , et al. Regulation by let‐7 and lin‐4 miRNAs results in target mRNA degradation. Cell. 2005;122(4):553‐563. 16122423 10.1016/j.cell.2005.07.031

[9] MicroRNAs BDP . genomics, biogenesis, mechanism, and function. Cell. 2004;116(2):281‐297. 14744438 10.1016/s0092-8674(04)00045-5

[10] Mitchell PS , Parkin RK , Kroh EM , et al. Circulating microRNAs as stable blood‐based markers for cancer detection. Proc Natl Acad Sci. 2008;105(30):10513‐10518. 18663219 10.1073/pnas.0804549105 PMC2492472

[11] Ghorbani S , Mahdavi R , Alipoor B , et al. Decreased serum microRNA‐21 level is associated with obesity in healthy and type 2 diabetic subjects. Arch Physiol Biochem. 2018;124(4):300‐305. 29113498 10.1080/13813455.2017.1396349

[12] Al‐Muhtaresh HA , Al‐Kafaji G . Evaluation of two‐diabetes related microRNAs suitability as earlier blood biomarkers for detecting prediabetes and type 2 diabetes mellitus. J Clin Med. 2018;7(2):12. 10.3390/jcm7020012 PMC5852428 29373500

[13] Su Y , Yuan J , Zhang F , et al. MicroRNA‐181a‐5p and microRNA‐181a‐3p cooperatively restrict vascular inflammation and atherosclerosis. Cell Death Dis. 2019;10(5):1‐15. 10.1038/s41419-019-1599-9 PMC6504957 31064980

[14] Kong L , Zhu J , Han W , et al. Significance of serum microRNAs in pre‐diabetes and newly diagnosed type 2 diabetes: a clinical study. Acta Diabetol. 2011;48(1):61‐69. 20857148 10.1007/s00592-010-0226-0

[15] Balasubramanyam M , Aravind S , Gokulakrishnan K , et al. Impaired miR‐146a expression links subclinical inflammation and insulin resistance in Type 2 diabetes. Mol Cell Biochem. 2011;351(1):197‐205. 21249428 10.1007/s11010-011-0727-3

[16] Karolina DS , Tavintharan S , Armugam A , et al. Circulating miRNA profiles in patients with metabolic syndrome. J Clin Endocrinol Metab. 2012;97(12):E2271‐E2276. 23032062 10.1210/jc.2012-1996

[17] Zhang T , Lv C , Li L , et al. Plasma miR‐126 is a potential biomarker for early prediction of type 2 diabetes mellitus in susceptible individuals. Biomed Res Int. 2013;2013:761617. 24455723 10.1155/2013/761617 PMC3886569

[18] Stępień EŁ , Durak‐Kozica M , Kamińska A , et al. Circulating ectosomes: determination of angiogenic microRNAs in type 2 diabetes. Theranostics. 2018;8(14):3874. 30083267 10.7150/thno.23334 PMC6071541

[19] Weale CJ , Matshazi DM , Davids SF , et al. Circulating miR‐30a‐5p and miR‐182‐5p in prediabetes and screen‐detected diabetes mellitus. Diabetes Metab Syndr Obes. 2020;13:5037. 33376373 10.2147/DMSO.S286081 PMC7762450

[20] Ju J , Xiao D , Shen N , et al. miR‐150 regulates glucose utilization through targeting GLUT4 in insulin‐resistant cardiomyocytes. Acta Biochim Biophys Sin. 2020;52(10):1111‐1119. 33085741 10.1093/abbs/gmaa094

[21] Massart J , Sjögren RJ , Lundell LS , et al. Altered miR‐29 expression in type 2 diabetes influences glucose and lipid metabolism in skeletal muscle. Diabetes. 2017;66(7):1807‐1818. 28404597 10.2337/db17-0141

[22] Shen Y , Xu H , Pan X , et al. miR‐34a and miR‐125b are upregulated in peripheral blood mononuclear cells from patients with type 2 diabetes mellitus. Exp Ther Med. 2017;14(6):5589‐5596. 29285097 10.3892/etm.2017.5254 PMC5740513

[23] Wang T‐T , Chen Y‐J , Sun L‐L , Zhang S‐J , Zhou Z‐Y , Qiao H . Affection of single‐nucleotide polymorphisms in miR‐27a, miR‐124a, and miR‐146a on susceptibility to type 2 diabetes mellitus in Chinese Han people. Chin Med J. 2015;128(4):533. 25673459 10.4103/0366-6999.151112 PMC4836260

[24] Zampetaki A , Kiechl S , Drozdov I , et al. Plasma microRNA profiling reveals loss of endothelial miR‐126 and other microRNAs in type 2 diabetes. Circ Res. 2010;107(6):810‐817. 20651284 10.1161/CIRCRESAHA.110.226357

[25] Olivieri F , Spazzafumo L , Bonafè M , et al. MiR‐21‐5p and miR‐126a‐3p levels in plasma and circulating angiogenic cells: relationship with type 2 diabetes complications. Oncotarget. 2015;6(34):35372. 26498351 10.18632/oncotarget.6164 PMC4742111

[26] Vatandoost N , Amini M , Iraj B , Momenzadeh S , Salehi R . Dysregulated miR‐103 and miR‐143 expression in peripheral blood mononuclear cells from induced prediabetes and type 2 diabetes rats. Gene. 2015;572(1):95‐100. 26164754 10.1016/j.gene.2015.07.015

[27] Sadeghzadeh S , Ashkezari MD , Seifati SM , et al. Circulating miR‐15a and miR‐222 as potential biomarkers of Type 2 diabetes. Diabetes Metab Syndr Obes. 2020;13:3461. 33061506 10.2147/DMSO.S263883 PMC7537850

[28] Shahrokhi SZ , Saeidi L , Sadatamini M , Jafarzadeh M , Rahimipour A , Kazerouni F . Can miR‐145‐5p be used as a marker in diabetic patients? Arch Physiol Biochem. 2020;1‐6. 10.1080/13813455.2020.1762657 32412315

[29] Higuchi C , Nakatsuka A , Eguchi J , et al. Identification of circulating miR‐101, miR‐375 and miR‐802 as biomarkers for type 2 diabetes. Metabolism. 2015;64(4):489‐497. 25726255 10.1016/j.metabol.2014.12.003

[30] Parrizas M , Mundet X , Castaño C , et al. miR‐10b and miR‐223‐3p in serum microvesicles signal progression from prediabetes to type 2 diabetes. J Endocrinol Invest. 2020;43(4):451‐459. 31721085 10.1007/s40618-019-01129-z

[31] Small EM , Olson EN . Pervasive roles of microRNAs in cardiovascular biology. Nature. 2011;469(7330):336‐342. 21248840 10.1038/nature09783 PMC3073349

[32] Bauersachs J , Thum T . Biogenesis and regulation of cardiovascular microRNAs. Circ Res. 2011;109(3):334‐347. 21778437 10.1161/CIRCRESAHA.110.228676

[33] Wang K , Zhang S , Weber J , Baxter D , Galas DJ . Export of microRNAs and microRNA‐protective protein by mammalian cells. Nucleic Acids Res. 2010;38(20):7248‐7259. 20615901 10.1093/nar/gkq601 PMC2978372

[34] Lv Y‐H , Ma J‐L , Pan H , et al. Estimation of the human postmortem interval using an established rat mathematical model and multi‐RNA markers. Forensic Sci Med Pathol. 2017;13(1):20‐27. 28032211 10.1007/s12024-016-9827-4

[35] Ying D , Yang SH , Sha L , et al. Circulating microRNAs as novel diagnostic biomarkers for very early‐onset (≤ 40 years) coronary artery disease. Biomed Environ Sci. 2016;29(8):545‐554. 27660218 10.3967/bes2016.073

[36] Faccini J , Ruidavets J‐B , Cordelier P , et al. Circulating miR‐155, miR‐145 and let‐7c as diagnostic biomarkers of the coronary artery disease. Sci Rep. 2017;7(1):1‐10. 28205634 10.1038/srep42916 PMC5311865

[37] Singh S , de Ronde MW , Kok MG , et al. MiR‐223‐3p and miR‐122‐5p as circulating biomarkers for plaque instability. Open Heart. 2020;7(1):e001223. 32487772 10.1136/openhrt-2019-001223 PMC7269547

[38] Hydbring P , Badalian‐Very G . Clinical applications of microRNAs. F1000Research. 2013;2:4‐19. 24627783 10.12688/f1000research.2-136.v1 PMC3917658

[39] Zhong Z , Hou J , Zhang Q , et al. Circulating microRNA expression profiling and bioinformatics analysis of dysregulated microRNAs of patients with coronary artery disease. Medicine. 2018;97(27):e11428. 29979444 10.1097/MD.0000000000011428 PMC6076134

[40] Li H , Zhang P , Li F , et al. Plasma miR‐22‐5p, miR‐132‐5p, and miR‐150‐3p are associated with acute myocardial infarction. Biomed Res Int. 2019;2019:5012648. 31179325 10.1155/2019/5012648 PMC6507259

[41] Xin Y , Yang C , Han Z . Circulating miR‐499 as a potential biomarker for acute myocardial infarction. Ann Transl Med. 2016;4(7):135. 27162785 10.21037/atm.2016.03.40 PMC4842393

[42] Corsten MF , Dennert R , Jochems S , et al. Circulating MicroRNA‐208b and MicroRNA‐499 reflect myocardial damage in cardiovascular disease. Circ Cardiovasc Genet. 2010;3(6):499‐506. 20921333 10.1161/CIRCGENETICS.110.957415

[43] Sun T , Dong Y‐H , Du W , et al. The role of microRNAs in myocardial infarction: from molecular mechanism to clinical application. Int J Mol Sci. 2017;18(4):745. 10.3390/ijms18040745 PMC5412330 28362341

[44] Liu Y , Li Q , Hosen MR , et al. Atherosclerotic conditions promote the packaging of functional microRNA‐92a‐3p into endothelial microvesicles. Circ Res. 2019;124(4):575‐587. 30582459 10.1161/CIRCRESAHA.118.314010

[45] Tang Y , Zhang Y , Chen Y , Xiang Y , Xie Y . Role of the micro RNA, miR‐206, and its target PIK 3C2α in endothelial progenitor cell function–potential link with coronary artery disease. FEBS J. 2015;282(19):3758‐3772. 26175229 10.1111/febs.13372

[46] Park E , Kim T‐H . Production of ABA responses requires both the nuclear and cytoplasmic functional involvement of PYR1. Biochem Biophys Res Comm. 2017;484(1):34‐39. 28109881 10.1016/j.bbrc.2017.01.082

[47] Libby P , Ridker PM , Hansson GK . Progress and challenges in translating the biology of atherosclerosis. Nature. 2011;473(7347):317‐325. 21593864 10.1038/nature10146

[48] Taurino C , Miller WH , McBride MW , et al. Gene expression profiling in whole blood of patients with coronary artery disease. Clin Sci. 2010;119(8):335‐343. 10.1042/CS20100043 PMC2922838 20528768

[49] Lin M , Zhang Y , Niu Z , Chi Y , Huang Q . Transcriptomic responses of peripheral blood cells to coronary artery disease. Bioscience Trends. 2018;12(4):354‐359. 30146615 10.5582/bst.2018.01078

[50] D'alessandra Y , Devanna P , Limana F , et al. Circulating microRNAs are new and sensitive biomarkers of myocardial infarction. Eur Heart J. 2010;31(22):2765‐2773. 20534597 10.1093/eurheartj/ehq167 PMC2980809

[51] Tutarel O , Dangwal S , Bretthauer J , et al. Circulating miR‐423_5p fails as a biomarker for systemic ventricular function in adults after atrial repair for transposition of the great arteries. Int J Cardiol. 2013;167(1):63‐66. 22188991 10.1016/j.ijcard.2011.11.082

[52] Kanuri SH , Kreutz RP . Micro RNA sequencing for myocardial infarction screening. Precision Medicine for Investigators, Practitioners and Providers: Elsevier; 2020:187‐198.

[53] Hullinger TG , Montgomery RL , Seto AG , et al. Inhibition of miR‐15 protects against cardiac ischemic injury. Circ Res. 2012;110(1):71‐81. 22052914 10.1161/CIRCRESAHA.111.244442 PMC3354618

[54] Vegter EL , Ovchinnikova ES , van Veldhuisen DJ , et al. Low circulating microRNA levels in heart failure patients are associated with atherosclerotic disease and cardiovascular‐related rehospitalizations. Clin Res Cardiol. 2017;106(8):598‐609. 28293796 10.1007/s00392-017-1096-z PMC5529487

[55] Jiménez‐Lucena R , Rangel‐Zúñiga OA , Alcalá‐Díaz JF , et al. Circulating miRNAs as predictive biomarkers of type 2 diabetes mellitus development in coronary heart disease patients from the CORDIOPREV study. Molecular therapy‐nucleic Acids. 2018;12:146‐157. 30195754 10.1016/j.omtn.2018.05.002 PMC6023857

[56] Voellenkle C , Van Rooij J , Cappuzzello C , et al. MicroRNA signatures in peripheral blood mononuclear cells of chronic heart failure patients. Physiol Genomics. 2010;42(3):420‐426. 20484156 10.1152/physiolgenomics.00211.2009

[57] Widera C , Gupta SK , Lorenzen JM , et al. Diagnostic and prognostic impact of six circulating microRNAs in acute coronary syndrome. J Mol Cell Cardiol. 2011;51(5):872‐875. 21806992 10.1016/j.yjmcc.2011.07.011

[58] Goren Y , Meiri E , Hogan C , et al. Relation of reduced expression of MiR‐150 in platelets to atrial fibrillation in patients with chronic systolic heart failure. Am J Cardiol. 2014;113(6):976‐981. 24462065 10.1016/j.amjcard.2013.11.060

[59] Fang ZY , Schull‐Meade R , Leano R , Mottram PM , Prins JB , Marwick TH . Screening for heart disease in diabetic subjects. Am Heart J. 2005;149(2):349‐354. 15846276 10.1016/j.ahj.2004.06.021

[60] Van Rooij E , Sutherland LB , Liu N , et al. A signature pattern of stress‐responsive microRNAs that can evoke cardiac hypertrophy and heart failure. Proc Natl Acad Sci. 2006;103(48):18255‐18260. 17108080 10.1073/pnas.0608791103 PMC1838739

[61] Weber M , Baker MB , Patel RS , Quyyumi AA , Bao G , Searles CD . MicroRNA expression profile in CAD patients and the impact of ACEI/ARB. Cardiol Res Pract. 2011;2011:532915. 21785714 10.4061/2011/532915 PMC3137987

[62] Sebastiani G , Nigi L , Spagnuolo I , Morganti E , Fondelli C , Dotta F . MicroRNA profiling in sera of patients with type 2 diabetes mellitus reveals an upregulation of miR‐31 expression in subjects with microvascular complications. J Biomed Sci Eng. 2013; 6:58‐64.

[63] Li X‐D , Yang Y‐J , Wang L‐Y , et al. Elevated plasma miRNA‐122,‐140‐3p,‐720,‐2861, and‐3149 during early period of acute coronary syndrome are derived from peripheral blood mononuclear cells. PLoS One. 2017;12(9):e0184256. 28961259 10.1371/journal.pone.0184256 PMC5621666

[64] Kuwabara Y , Ono K , Horie T , et al. Increased microRNA‐1 and microRNA‐133a levels in serum of patients with cardiovascular disease indicate myocardial damage. Circ Cardiovasc Genet. 2011;4(4):446‐454. 21642241 10.1161/CIRCGENETICS.110.958975

[65] Fichtlscherer S , De Rosa S , Fox H , et al. Circulating microRNAs in patients with coronary artery disease. Circ Res. 2010;107(5):677‐684. 20595655 10.1161/CIRCRESAHA.109.215566

[66] Diehl P , Fricke A , Sander L , et al. Microparticles: major transport vehicles for distinct microRNAs in circulation. Cardiovasc Res. 2012;93(4):633‐644. 22258631 10.1093/cvr/cvs007 PMC3291092

[67] Li T , Cao H , Zhuang J , et al. Identification of miR‐130a, miR‐27b and miR‐210 as serum biomarkers for atherosclerosis obliterans. Clin Chim Acta. 2011;412(1–2):66‐70. 20888330 10.1016/j.cca.2010.09.029

[68] Hoekstra M , van der Lans CA , Halvorsen B , et al. The peripheral blood mononuclear cell microRNA signature of coronary artery disease. Biochem Biophys Res Comm. 2010;394(3):792‐797. 20230787 10.1016/j.bbrc.2010.03.075

[69] Takahashi Y , Satoh M , Minami Y , Tabuchi T , Itoh T , Nakamura M . Expression of miR‐146a/b is associated with the Toll‐like receptor 4 signal in coronary artery disease: effect of renin–angiotensin system blockade and statins on miRNA‐146a/b and Toll‐like receptor 4 levels. Clin Sci. 2010;119(9):395‐405. 10.1042/CS20100003 20524934

[70] Lew JKS , Pearson JT , Schwenke DO , Katare R . Exercise mediated protection of diabetic heart through modulation of microRNA mediated molecular pathways. Cardiovasc Diabetol. 2017;16(1):10. 28086863 10.1186/s12933-016-0484-4 PMC5237289

[71] Rawal S , Manning P , Katare R . Cardiovascular microRNAs: as modulators and diagnostic biomarkers of diabetic heart disease. Cardiovasc Diabetol. 2014;13(1):1‐24. 24528626 10.1186/1475-2840-13-44 PMC3976030

[72] Yaghoubi A , Ghojazadeh M , Abolhasani S , Alikhah H , Khaki‐Khatibi F . Correlation of serum levels of vitronectin, malondialdehyde and Hs‐CRP with disease severity in coronary artery disease. J Cardiovasc Thorac Res. 2015;7(3):113. 26430499 10.15171/jcvtr.2015.24 PMC4586597

[73] Khaki‐Khatibi F , Mahmoodi N , Abolhasani S , Gharebaba RP . Correlation of fibrinolysis marker of plasma plasminogen activator inhibitor type‐1 and oxidative stress parameters in occurrence and progression of coronary artery disease. Bull Env Pharmacol Life Sci. 2014;3(7):27‐33.

[74] Mahmoodi N , Abolhasani S . Relationship between serum levels of fibronectin, leptin and hs‐CRP with extension of coronary artery disease. Bull Env Pharmacol Life Sci. 2014;3:40‐46.

[75] Khanbabaei N , Saadati HM , Shahbazloo SV , et al. Association of serum leptin with angiographically proven cardiovascular disease and with components of the metabolic syndrome: a cross‐sectional study in East Azerbaijan. Cardiovasc Endocrinol Metab. 2021;10(1):45. 33634255 10.1097/XCE.0000000000000227 PMC7901824

[76] Ramzan F , D’Souza R , Durainayagam B , et al. Circulatory miRNA biomarkers of metabolic syndrome. Acta Diabetol. 2020;57(2):203‐214. 31435783 10.1007/s00592-019-01406-6

[77] Deng X , Liu Y , Luo M , et al. Circulating miRNA‐24 and its target YKL‐40 as potential biomarkers in patients with coronary heart disease and type 2 diabetes mellitus. Oncotarget. 2017;8(38):63038. 28968969 10.18632/oncotarget.18593 PMC5609901

[78] Jimenez‐Lucena R , Alcala‐Diaz JF , Roncero‐Ramos I , et al. MiRNAs profile as biomarkers of nutritional therapy for the prevention of Type 2 diabetes mellitus: From the CORDIOPREV study. Clin Nutr. 2021;40(3):1028‐1038. 32723508 10.1016/j.clnu.2020.06.035

[79] Costantino S , Paneni F , Lüscher TF , Cosentino F . MicroRNA profiling unveils hyperglycaemic memory in the diabetic heart. Eur Heart J. 2015;37(6):572‐576. 26553540 10.1093/eurheartj/ehv599

[80] Wang W , Li Z , Zheng Y , Yan M , Cui Y , Jiang J . Circulating microRNA‐92a level predicts acute coronary syndrome in diabetic patients with coronary heart disease. Lipids Health Dis. 2019;18(1):1‐8. 30670045 10.1186/s12944-019-0964-0 PMC6343303

[81] Ruiz MA , Chakrabarti S . MicroRNAs: the underlying mediators of pathogenetic processes in vascular complications of diabetes. Can J Diabetes. 2013;37(5):339‐344. 24500562 10.1016/j.jcjd.2013.07.003

[82] Muñoz JP , Collao A , Chiong M , et al. The transcription factor MEF2C mediates cardiomyocyte hypertrophy induced by IGF‐1 signaling. Biochem Biophys Res Comm. 2009;388(1):155‐160. 19654000 10.1016/j.bbrc.2009.07.147

[83] Katayama M , Wiklander OP , Fritz T , et al. Circulating exosomal miR‐20b‐5p is elevated in type 2 diabetes and could impair insulin action in human skeletal muscle. Diabetes. 2019;68(3):515‐526. 30552111 10.2337/db18-0470

[84] Mensà E , Giuliani A , Matacchione G , et al. Circulating miR‐146a in healthy aging and type 2 diabetes: age‐and gender‐specific trajectories. Mech Ageing Dev. 2019;180:1‐10. 30880174 10.1016/j.mad.2019.03.001

[85] Aronica E , Fluiter K , Iyer A , et al. Expression pattern of miR‐146a, an inflammation‐associated microRNA, in experimental and human temporal lobe epilepsy. Eur J Neurosci. 2010;31(6):1100‐1107. 20214679 10.1111/j.1460-9568.2010.07122.x

[86] De Rosa S , Arcidiacono B , Chiefari E , Brunetti A , Indolfi C , Foti DP . Type 2 diabetes mellitus and cardiovascular disease: genetic and epigenetic links. Front Endocrinol. 2018;9:2. 10.3389/fendo.2018.00002 PMC5776102 29387042

[87] Yildirim SS , Akman D , Catalucci D , Turan B . Relationship between downregulation of miRNAs and increase of oxidative stress in the development of diabetic cardiac dysfunction: junctin as a target protein of miR‐1. Cell Biochem Biophys. 2013;67(3):1397‐1408. 23723006 10.1007/s12013-013-9672-y

[88] Pan Z , Sun X , Ren J , et al. miR‐1 exacerbates cardiac ischemia‐reperfusion injury in mouse models. PLoS One. 2012;7(11):e50515. 23226300 10.1371/journal.pone.0050515 PMC3511560

[89] Fan Z , Peng W , Wang Z , Zhang L , Liu K . Identification of biomarkers associated with metabolic cardiovascular disease using mRNA‐SNP‐miRNA regulatory network analysis. BMC Cardiovasc Disord. 2021;21(1):1‐12. 34301176 10.1186/s12872-021-02166-4 PMC8305867

[90] Ding Y , Sun X , Shan P‐F . MicroRNAs and cardiovascular disease in diabetes mellitus. Biomed Res Int. 2017;2017. 10.1155/2017/4080364 PMC5337313 28299324

[91] Petrie JR , Guzik TJ , Touyz RM . Diabetes, hypertension, and cardiovascular disease: clinical insights and vascular mechanisms. Can J Cardiol. 2018;34(5):575‐584. 29459239 10.1016/j.cjca.2017.12.005 PMC5953551

[92] Yamada H , Suzuki K , Fujii R , et al. Circulating miR‐21, miR‐29a, and miR‐126 are associated with premature death risk due to cancer and cardiovascular disease: the JACC Study. Sci Rep. 2021;11(1):1‐9. 33674633 10.1038/s41598-021-84707-7 PMC7935984

[93] Raitoharju E , Lyytikäinen L‐P , Levula M , et al. miR‐21, miR‐210, miR‐34a, and miR‐146a/b are up‐regulated in human atherosclerotic plaques in the tampere vascular study. Atherosclerosis. 2011;219(1):211‐217. 21820659 10.1016/j.atherosclerosis.2011.07.020

[94] Hromadka M , Motovska Z , Hlinomaz O , et al. MiR‐126‐3p and MiR‐223‐3p as biomarkers for prediction of thrombotic risk in patients with acute myocardial infarction and primary angioplasty. J Pers Med. 2021;11(6):508. 34199723 10.3390/jpm11060508 PMC8230013

[95] Li X , Yao Q , Cui H , et al. MiR‐223 or miR‐126 predicts resistance to dual antiplatelet therapy in patients with ST‐elevation myocardial infarction. J Int Med Res. 2021;49(6):03000605211016209. 10.1177/03000605211016209 PMC8191085 34098766

[96] Li C , Chen X , Huang J , Sun Q , Wang L . Clinical impact of circulating miR‐26a, miR‐191, and miR‐208b in plasma of patients with acute myocardial infarction. Eur J Med Res. 2015;20(1):1‐8. 26044724 10.1186/s40001-015-0148-y PMC4459687

[97] Archer K , Broskova Z , Bayoumi AS , et al. Long non‐coding RNAs as master regulators in cardiovascular diseases. Int J Mol Sci. 2015;16(10):23651‐23667. 26445043 10.3390/ijms161023651 PMC4632719

[98] Siasos G , Bletsa E , Stampouloglou PK , et al. MicroRNAs in cardiovascular disease. Hell J Cardiol. 2020;61(3):165‐173. 10.1016/j.hjc.2020.03.003 32305497

[99] Paquissi FC . The role of inflammation in cardiovascular diseases: the predictive value of neutrophil‐lymphocyte ratio as a marker in peripheral arterial disease. Ther Clin Risk Manag. 2016;12:851‐860. 27313459 10.2147/TCRM.S107635 PMC4892833

[100] Gregersen I , Halvorsen B . Inflammatory mechanisms in atherosclerosis. Atherosclerosis‐Yesterday: Today and Tomorrow; 2018:10.

[101] Wang W , Zheng Y , Li Z , Cui Y , Jiang J . MiR‐92a contributes to the cardiovascular disease development in diabetes mellitus through NF‐κB and downstream inflammatory pathways. Eur Rev Med Pharmacol Sci. 2019;23(7):3070‐3079. 31002156 10.26355/eurrev_201904_17589

[102] Zhang L , Huang D , Wang Q , et al. MiR‐132 inhibits expression of SIRT1 and induces pro‐inflammatory processes of vascular endothelial inflammation through blockade of the SREBP‐1c metabolic pathway. Cardiovasc Drugs Ther. 2014;28(4):303‐311. 24924687 10.1007/s10557-014-6533-x

[103] Šatrauskienė A , Navickas R , Laucevičius A , et al. Mir‐1, miR‐122, miR‐132, and miR‐133 are related to subclinical aortic atherosclerosis associated with metabolic syndrome. Int J Environ Res Public Health. 2021;18(4):1483. 33557426 10.3390/ijerph18041483 PMC7915826

[104] Montgomery RL , Hullinger TG , Semus HM , et al. Therapeutic inhibition of miR‐208a improves cardiac function and survival during heart failure. Circulation. 2011;124(14):1537‐1547. 21900086 10.1161/CIRCULATIONAHA.111.030932 PMC3353551

